# Superradiant terahertz free-electron laser driven by electron microbunch trains

**DOI:** 10.1038/s41377-025-02156-7

**Published:** 2026-01-08

**Authors:** Yifan Liang, Tong Li, Jitao Sun, Zhuoyuan Liu, Jiayue Yang, Xiaofan Wang, Yong Yu, Qili Tian, Zhigang He, Hongfei Wang, Li Zeng, Huaiqian Yi, Hao Sun, Yingjie Dai, Xiujie Deng, Guorong Wu, Weiqing Zhang, Xueming Yang, Chuanxiang Tang, Lixin Yan

**Affiliations:** 1 Institute of Advanced Light Source Facilities, Shenzhen, 518107 China; 2 Department of Engineering Physics, Tsinghua University, Beijing, 100084 China; 3 Dalian Institute of Chemical Physics, CAS, Dalian, 116023 China

**Keywords:** Free-electron lasers, Terahertz optics

## Abstract

Superradiance, an enhanced radiation phenomenon stemming from the phase synchronization of emitters, features a radiation intensity proportional to the number of emitters squared. The pursuit of superradiance from free electrons has long been a goal for generating intense radiation across a broad spectrum, from terahertz (THz) to the X-ray regime. However, achieving superradiance in the THz spectral range has been hindered by the lack of effective microbunching techniques. Here, we demonstrate an ultra-widely tunable superradiant THz free-electron laser (FEL) driven by high-peak-current electron microbunch trains. The emission efficiency is substantially improved as the ultra-short electron microbunches emit in phase and engage in strong interactions with the generated THz waves within the undulator. We further demonstrate that the implementation of a tapered undulator configuration leads to a two-fold enhancement in emission intensity compared to the non-tapered case, elevating the pulse energy of the narrow-band THz radiation to the millijoule level in a one-meter-long undulator. This experimental breakthrough represents a critical step toward realizing a compact, high-power, narrow-band THz source capable of fully bridging the ‘THz gap’ and will unlock numerous opportunities across a wide range of scientific disciplines.

## Introduction

The THz radiation, situated between microwaves and infrared light, has become a transformative tool for scientific and technological advancements. This unique radiation regime enables direct access to key low-energy excitations in condensed matter systems, including lattice vibrations (phonons) governing structural dynamics^1,2^, collective spin excitations (magnons) in magnetic materials^3,4^, and coherent quasiparticle interactions in correlated electron systems^5,6^. Moreover, intense THz fields have demonstrated remarkable capabilities in driving non-perturbative nonlinearities, such as field-induced phase transitions^7^, coherent interband polarization^8^ and large-amplitude spin oscillations^9^. However, the full exploitation of THz photonics has been fundamentally constrained by the absence of a versatile radiation source combining high peak power, narrow spectral bandwidth, and broad tunability in a compact architecture.

Current state-of-the-art THz sources still face limitations in frequency tunability or pulse energy. FELs have the potential to deliver high pulse energy and offer wide frequency tunability. In FELs, light amplification occurs when an ultra-relativistic electron beam, guided by the static, periodic magnetic field of an undulator, resonantly interacts with a co-propagating electromagnetic wave. Conventional THz FELs typically operate in the low-gain oscillator mode^10–14^ which limits the energy extraction efficiency and peak power. In recent years, with the development of high-brightness electron sources, single-pass THz FELs have emerged where beams with very high charge are utilized to self-amplify the incoherent spontaneous emission from shot noise (SASE) over a long undulator^15^. The radiation power and energy extraction efficiency are constrained due to the temporal and spatial walk-off, namely slippage and diffraction, between the radiation and the electron bunch. To mitigate the slippage and diffraction effects, waveguides could be implemented^16^, but the frequency tuning range is limited^17^. Another category of free-electron-driven THz sources exploits sub-picosecond electron bunches^18^. However, stringent requirements on the bunch length and the beam charge limit both the pulse energy and the tunability.

To overcome these limitations, a compelling mechanism is the superradiant THz FEL where the electron beams are pre-modulated into periodically spaced microbunches with lengths much shorter than the radiation wavelength. In this way, the microbunching process in classical high-gain FEL is bypassed, and the emissions from electrons add in phase, producing coherent radiation with intensity scaling quadratically with the number of electrons. As the beam propagates and evolves along the undulator, the electrons interact with the fields generated by the trailing microbunches and further amplify the radiation to saturation. By circumventing the exponential gain process in high-gain FELs, the electrons are initially trapped at the maximum decelerating phase within a larger ponderomotive bucket formed by the strong coherent field, thereby enabling higher saturated radiation energy in a much shorter saturation length^19^. The superradiant FEL thus offers a distinct pathway to high-intensity coherent radiation with enhanced efficiency.

Despite the theoretical advantages, the experimental realization of superradiant FELs in the THz regime has been fraught with difficulties due to the lack of effective microbunching techniques. Unlike the optical spectrum, where a wide range of well-developed laser technologies are available to modulate the electron beams, the THz region lacks comparable sources with the necessary characteristics, such as sufficient intensity and appropriate frequency tunability. Although there have been advances in accelerator-based methods for generating THz-scale electron microbunch trains^20–30^, challenges in achieving wide frequency tunability and high bunching factor persist. Addressing these challenges is critical for advancing THz FELs as versatile, high-performance radiation sources for scientific and industrial applications.

Here we report the experimental demonstration of a superradiant THz FEL driven by deeply modulated electron microbunch trains. The microbunching scheme adopted in our research entails the conversion of the steep energy modulation, engendered by the nonlinear longitudinal space-charge (NLSC) force, into a highly pronounced density modulation^31^. The beam was then sent into a compact undulator (20 periods, 1 m total length) to drive the superradiant FEL. By implementing a harmonic cavity for beam energy chirp linearization, we achieved continuous frequency tuning across the 1–20 THz spectral range. Furthermore, we demonstrate that incorporating a tapered undulator configuration further enhanced the emission performance, pushing narrowband THz pulse energies to the millijoule (mJ) level.

## Results

### Superradiant emission

**Fig. 1:**
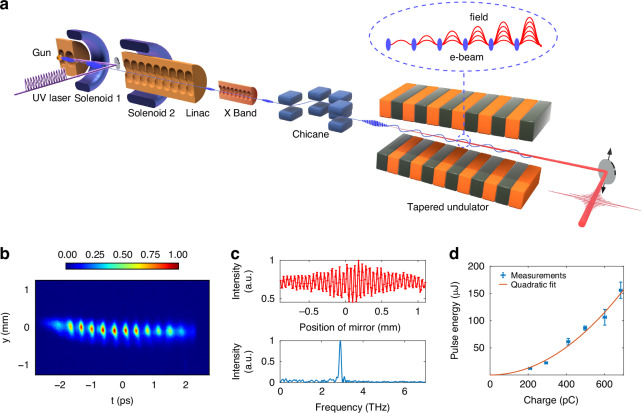
Schematic layout of the superradiant THz FEL experiment. **a** The photocathode was illuminated with laser pulse trains to impose initial density modulation on the electron beam. The longitudinal space-charge force converted this initial density modulation into energy modulation, which reverted to shallow density spikes before injection into the linac. During subsequent acceleration and drift sections, space-charge effects from density spikes induced sharp energy modulation, which was transformed into deep density modulation by the chicane. The beam was then sent into a 20-period undulator to drive the superradiant THz FEL, where the THz fields emitted by the microbunches coherently added. **b** Measured longitudinal distribution of the electron microbunch train. The calibrated bunching frequency is $2.82\pm 0.07$THz. **c** A typical autocorrelation function and its Fourier transform of FEL pulses. The central frequency lies at 2.9 THz. **d** The relationship between the THz pulse energy and the bunch charge at the corresponding emission frequency shown in (**c**). The blue dots represent the experimental data and the red curve is a quadratic fit. The error bars in pulse energy measurements represent root mean square intensity fluctuations of 100 shots

Our experiment was conducted at the Dalian Coherent Light Source (DCLS)^32^, with the schematic layout illustrated in Fig. 1a. The physical process began with the illumination of the photocathode by laser pulse trains, which imposed an initial periodic density modulation on the electron beam. This initial longitudinal density modulation gave rise to a corresponding periodic longitudinal space charge force, which converted the density modulation into the energy modulation. Through precise control of the beam focus, the energy modulation transformed back into shallow density spikes before the beam entered the linear accelerator (linac). The linac accelerated the beam to the undulator resonant energy while simultaneously imposing an energy chirp on the beam by setting the acceleration phase off-crest. To linearize the beam energy chirp, a harmonic cavity was employed downstream of the linac. As the beam drifted from the linac entrance to the chicane entrance, the longitudinal space charge force arising from the periodic density spikes induced a sharp energy modulation on the beam. This energy modulation was subsequently converted into a deep density modulation after the beam passed through a dispersive section (a magnetic chicane).

In Fig. 1b, we present a typical longitudinal distribution of the microbunch trains measured by a deflector at the end of the beamline. The results clearly show that the longitudinal lengths of the discrete microbunches are much shorter than their distribution periods, confirming the generation of microbunch trains with well-defined spacing. This precise control over the beam’s longitudinal structure is critical for achieving superradiance at terahertz frequencies.

The generated electron microbunch trains were injected into a 20-period undulator to drive the superradiant THz FEL. The undulator magnetic field strength *B*_0_ was fine-tuned to satisfy the resonance condition ${f}_{r}=\frac{2c{\gamma }^{2}}{{\lambda }_{U}(1+{K}^{2}/2)}$, where ${f}_{r}$ is the resonant frequency, *c* is the speed of light in free space, $\gamma$ is the beam relativistic factor, ${\lambda }_{U}$ is the undulator period length, $K=e{B}_{0}{\lambda }_{U}/2\pi {m}_{e}c$ is the undulator parameter where *e* is the electron charge and ${m}_{e}$ is the electron mass. By systematically adjusting the undulator magnetic field strength, a pronounced increase in pulse energy was observed when the resonant frequency matched the microbunch spacing, confirming the coherent enhancement of radiation emission.

In Fig. 1c, the autocorrelation of the FEL pulse and its corresponding Fourier transform are presented, demonstrating the narrowband spectral characteristics of the emitted radiation. Furthermore, Fig. 1d illustrates the relationship between the pulse energy and the bunch charge at the frequency shown in Fig. 1c, revealing a quadratic dependence that aligns perfectly with the theoretical predictions for superradiant emission. This quadratic scaling, combined with the narrowband nature of the radiation, provides definitive evidence of superradiant emission originating from the coherent interaction between microbunches and the electromagnetic wave within the undulator.

### Frequency tuning

**Fig. 2:**
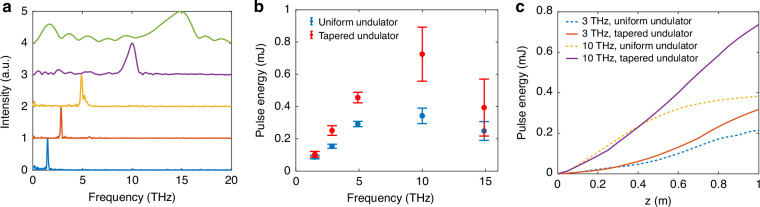
Measurements of THz FEL spectrum and pulse energy. **a** Measured THz FEL spectrum from the Fourier transform of the autocorrelation function. The frequency was tuned by changing the microbunch spacing and the undulator resonant frequency simultaneously. **b** Measured THz pulse energy at different frequencies without (blue dots) and with (red dots) undulator tapering. The error bars represent root mean square intensity fluctuations of 100 shots. **c** Simulated THz energy growth along the undulator axis for both uniform (dashed lines) and tapered (solid lines) configurations at 3 THz and 10 THz

By adjusting the longitudinal energy chirp of the entire electron bunch and the magnetic chicane strength, the electron beam was dynamically stretched or compressed, enabling continuous tuning of the bunching frequency across a broad range from 1 THz to 15 THz. The spectra of THz FEL pulses are shown in Fig. 2a. To achieve higher bunching frequencies, we increased the beam energy chirp and implemented beam compression. Although the X-band cavity effectively linearized the primary energy chirp, residual microbunch spacing variations persisted due to higher-order chirp components induced by space charge effects. Consequently, a higher bunching frequency was associated with a broader linewidth and a more significant beam energy spread. Since the FEL performance is intrinsically linked to the beam qualities, the linewidth is broadened across the tuning range, as depicted in Fig. 2a.

For each FEL pulse, the pulse energy was measured by inserting a THz attenuator and a filter before the detector (see Methods). Figure 2b presents the measured FEL pulse energy across the operational frequency range. In our scheme, as the frequency was tuned from 1 THz to 15 THz, the microbunch length underwent compression while maintaining a high bunching factor. The linewidth broadening during this process, combined with the pulse energy’s dependence on both photon energy and the square of the bunching factor, resulted in the maximum pulse energy at approximately 10 THz. At frequencies beyond 10 THz, a slight decrease in pulse energy was observed, which can be attributed to the gradual reduction in bunching factor caused by nonlinear compression dynamics.

### Tapering enhancement

After the electrons exchange a substantial amount of energy with the electromagnetic wave, they begin to deviate from the resonance, leading to saturation of the radiation emission. This issue can be mitigated by tapering the undulator, which maintains the resonance of the electrons despite their energy loss during the interaction with the radiation field. For our tapered undulator, the magnetic field *B*(z) as a function of the longitudinal position *z* can be described by *B*(z) = *B*_0_(1 + *αz*), where *B*_0_ is the initial magnetic field strength at z = 0, and *α* is the tapering parameter.

As illustrated in Fig. 2b, the implementation of undulator tapering resulted in a 20% to 110% enhancement in pulse energy across the frequency range from 1.5 THz to 15 THz. This frequency-dependent variation in tapering efficiency stems primarily from the interplay between diffraction effects and beam-wave interaction efficiency—with longer wavelengths exhibiting more pronounced diffraction losses that degrade coupling efficiency within the undulator.

Numerical simulations employing the three-dimensional time-dependent FEL code Genesis^33^ reveal distinct saturation dynamics for different operational frequencies. Figure 2c demonstrates the comparative evolution of pulse energy along the undulator axis for both uniform and tapered configurations at 3 THz and 10 THz. Notably, while the 3 THz radiation fails to reach saturation within the 20-period undulator, the 10 THz emission is close to saturation near the undulator exit. This spatial disparity in saturation points directly correlates with the observed enhancement factors in the tapered configuration, explaining the improved tapering efficiency at higher frequencies where sustained strong interaction strength enables more efficient energy extraction.

### Harmonic generation

**Fig. 3:**
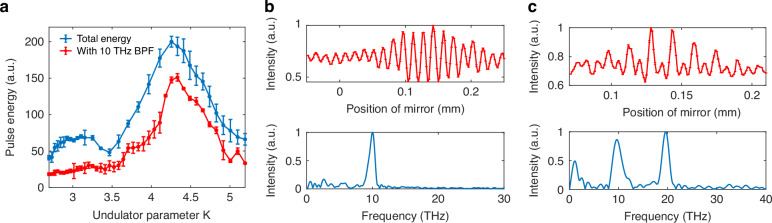
Measurements of the second harmonic. **a** Scanning the pulse energy by varying the undulator parameter. The blue solid line represents the measurement results obtained without any filter, while the red dashed line corresponds to measurements with a 10 THz band-pass filter. The pulse energy has been calibrated by considering the BPF transmission efficiency. The error bars represent root mean square intensity fluctuations of 20 shots. Autocorrelation functions and their Fourier transforms measured without any filter when the undulator parameter was (**b**) 4.3 (resonant at 10 THz) and (**c**) 2.9 (resonant at 20 THz)

Due to the significantly shorter length of the generated microbunches compared to the spacing between them, the electron microbunch trains exhibited the capability to emit radiation at their second harmonic when the beam’s resonant frequency in the undulator was tuned to twice the bunching frequency. To illustrate this phenomenon, we conducted experiments using a beam with a 10 THz modulation frequency. By systematically scanning the undulator parameter, we measured both the total pulse energy and the pulse energy filtered through a 10 THz band-pass filter (BPF). The results, as depicted in Fig. 3a, revealed a prominent energy peak when the resonant frequency was tuned to approximately 10 THz, corresponding to the fundamental microbunching frequency. Additionally, a smaller energy hump was observed when the resonant frequency was adjusted to the second harmonic frequency at 20 THz. The radiation energy of this second harmonic was noticeably weaker, consistent with the lower bunching factor at 20 THz compared to that at the fundamental frequency of 10 THz.

To further characterize the spectral properties, we measured the autocorrelation function without any spectral filtering and obtained the corresponding spectra for two distinct undulator parameter settings: 4.3 (resonant at 10 THz) and 2.9 (resonant at 20 THz). The results, presented in Fig. 3b, c, demonstrate that when the undulator resonant frequency matched the microbunching frequency (10 THz), the spectrum exhibited a single dominant peak at 10 THz. In contrast, when the resonant frequency was tuned to the second harmonic (20 THz), a distinct 20 THz component emerged in the spectrum, confirming the efficient emission of the second harmonic radiation. Owing to the strong bunching characteristics of the compressed and modulated beam at frequencies below 1 THz and around 10 THz, we attribute both spectral features in Fig. 3c - the low-frequency component and the 10 THz peak - to the combined contributions of coherent synchrotron radiation from the undulator and coherent transition radiation at the coupling mirror.

## Discussion

To summarize, we have successfully demonstrated a superradiant THz free-electron laser driven by deeply modulated electron microbunch trains. This compact system exhibits unprecedented performance, delivering ultra-wide frequency tunability (1–20 THz) and high pulse energy (reaching millijoule levels) when driven by a 700-pC electron beam at tens of MeV energy. In this work, the undulator has a maximum undulator parameter K of 5.6 and the lowest beam relativistic factor is about 36 (~18 MeV), which limits the lowest frequency to 1 THz. Since the microbunching frequency can be tuned as low as 0.1 THz, by replacing the undulator with one featuring a 10 cm period length, the radiation frequency can be extended down to 0.1 THz.

Our scheme achieves what was previously unattainable: mJ-level pulse energies combined with narrow-bandwidth emission across an exceptionally wide tuning range. This represents a 2-3 orders of magnitude improvement in pulse energy for broadly tunable narrow-bandwidth sources compared to current state-of-the-art technologies. Our work bridges the critical “THz gap” with a robust, accelerator-based solution that combines high pulse energy, exceptional spectral control, and scalability. The system’s compact footprint and reliance on well-developed technologies make it readily deployable for applications ranging from nonlinear THz spectroscopy to quantum material control and novel THz device development^34,35^. This breakthrough establishes a new paradigm for on-demand, high-intensity THz sources, unlocking transformative opportunities across physics, chemistry, and materials science.

## Materials and methods

### DCLS beamline

The layout of the DCLS beamline is illustrated in extended Data Fig. 1. A 700-pC electron beam was generated by a radio-frequency photocathode gun and accelerated to 5 MeV before injection into three S-band linacs. Two solenoids were employed for beam focusing, with one positioned at the gun exit and the other along the linac. The first linac was operated on-crest, accelerating the beam to approximately 51 MeV, while the subsequent two linacs were operated off-crest to impose a negative or positive energy chirp on the electron beam, decelerating or accelerating it to the undulator resonant energy (18 MeV–55 MeV, corresponding to 1 THz–15 THz). The peak field strengths in these linacs were precisely tuned to control the energy chirp amplitude and compression rate. A fourth-harmonic X-band cavity was utilized to linearize the energy chirp, ensuring optimal beam quality. The beam then traversed a magnetic chicane, where it was either stretched or compressed depending on the applied energy chirp rate.

Following the chicane, a planar undulator with 20 periods and a 5 cm period length was installed. The undulator gap was adjustable from 60 mm to 9 mm, enabling tuning of the undulator parameter from 0 to 5.6. A linear taper was applied to the undulator gap, resulting in a corresponding linear taper of the magnetic field along the undulator. The tapering parameter α was tuned from 0 to 0.3 in the experiment to optimize the pulse energy. By gradually increasing the tapering parameter α, we observed an increase and then a decrease in pulse energy in both the simulation and the experiment. The optimized tapering parameter was obtained when the pulse energy reached its maximum.

Subsequently, the beam was further accelerated by four additional linacs to 255 MeV, with the energy chirp compensated. After drifting through an additional 50 meters, the beam was diagnosed using a deflecting cavity and a dipole magnet. Since the beam energy is sufficiently high to fix the longitudinal distribution and experiences little dispersion during its transit to the end of the beamline, the measured temporal profile is nearly identical to the beam’s appearance at the entrance of the undulator.

### Photoinjector drive laser generation and measurements

The drive laser for the photoinjector was generated by a frequency-tripled Ti:sapphire laser system, as shown in extended data Fig. 2. The 260-nm ultraviolet (UV) pulse was directed into four alpha-barium borates (α-BBO) crystals to produce a laser pulse train consisting of 16 pulses equally spaced at intervals of 0.5 ps. To equivalently measure the laser longitudinal profile on the photocathode, a movable mirror was utilized to switch the UV pulse train onto the measurement path, which replicated the actual transportation path to the photocathode with the same optics and distance. An infrared (IR) pulse with a duration of about 50 fs temporarily swept over the UV train inside a 0.1-mm-thick β-BBO crystal, producing a difference-frequency signal that was then detected by a photomultiplier tube (PMT). The full width at half maximum (FWHM) of the single UV pulse was measured to be about 140 fs.

### THz measurements

The THz measurement platform was mounted immediately after the undulator exit. As illustrated in the extended data Fig. 3, the THz radiation reflected out of the vacuum chamber through a diamond window was collected by two gold-coated off-axis mirrors (OAPs). A flipping mirror switched the THz radiation directly to a Golay cell (TYDEX, model GC-1D), or to a Michelson interferometer. The Golay cell detector was calibrated to a sensitivity of 1.8 μJ/V using a CO_2_ laser operating at a wavelength of 10.6 μm. A THz attenuator with a transmission efficiency of 1%, along with either a band-pass filter (BPF) or a low-pass filter (LPF), was positioned in front of the detector. Initially, the THz pulse spectrum was measured using a Michelson interferometer to determine the radiation frequency, followed by measurements of the pulse energy. For frequencies of 1.5 THz, 3 THz, 5 THz, 10 THz, and 15 THz, BPFs centered at the respective frequencies were placed before the Golay cell to measure the energy with and without the undulator tapering. In the case of the tapering configuration at 10 THz, the spectral width underwent broadening as a result of the tapering process, extending beyond the pass-band of the 10 THz BPF. We used a LPF with a cutoff frequency of 5.5 THz to measure the energy contribution from frequencies below 5.5 THz. The radiation energy around the 10 THz frequency peak was then calculated by subtracting the energy below 5.5 THz from the total energy measured in the absence of any filters. All reported energy measurements have considered the transmission efficiencies of the attenuator, filters, and the vacuum window.

### Simulation method

The particle-tracking code ASTRA^36^ and ELEGANT^37^ were used to simulate the beam dynamics from the photocathode to the end of the beamline with the space charge effects considered. The time-dependent FEL code Genesis was utilized to simulate the FEL from THz electron microbunch trains. The 6D beam distribution from the ELEGANT simulation was directly imported into Genesis and used to load the phase space. The space charge effects and the diffraction effects were both considered in the FEL simulation.

## Supplementary information

Extended Data

## Data Availability

The data that support the findings of this study are available from the corresponding author on reasonable request.
