# Supplementary material for: Superradiant terahertz free-electron laser driven by electron microbunch trains
Source: Light Sci Appl. 2026 Jan 8;15:60. doi: 10.1038/s41377-025-02156-7 (PMC12779983; doi:10.1038/s41377-025-02156-7)
Supplement: Supplementary file 1 — Extended Data [file 41377_2025_2156_MOESM1_ESM.docx]

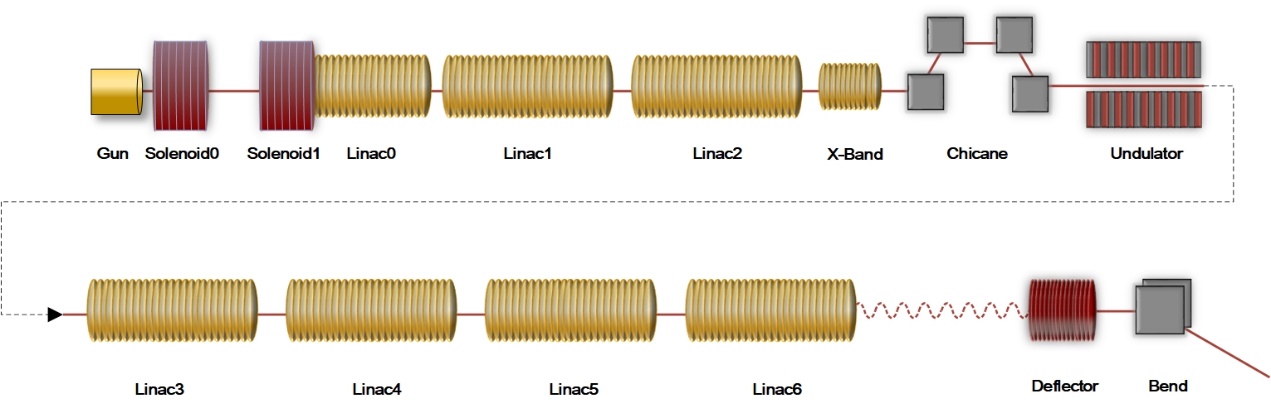


**Extended Data Fig. 1 DCLS beamline.**


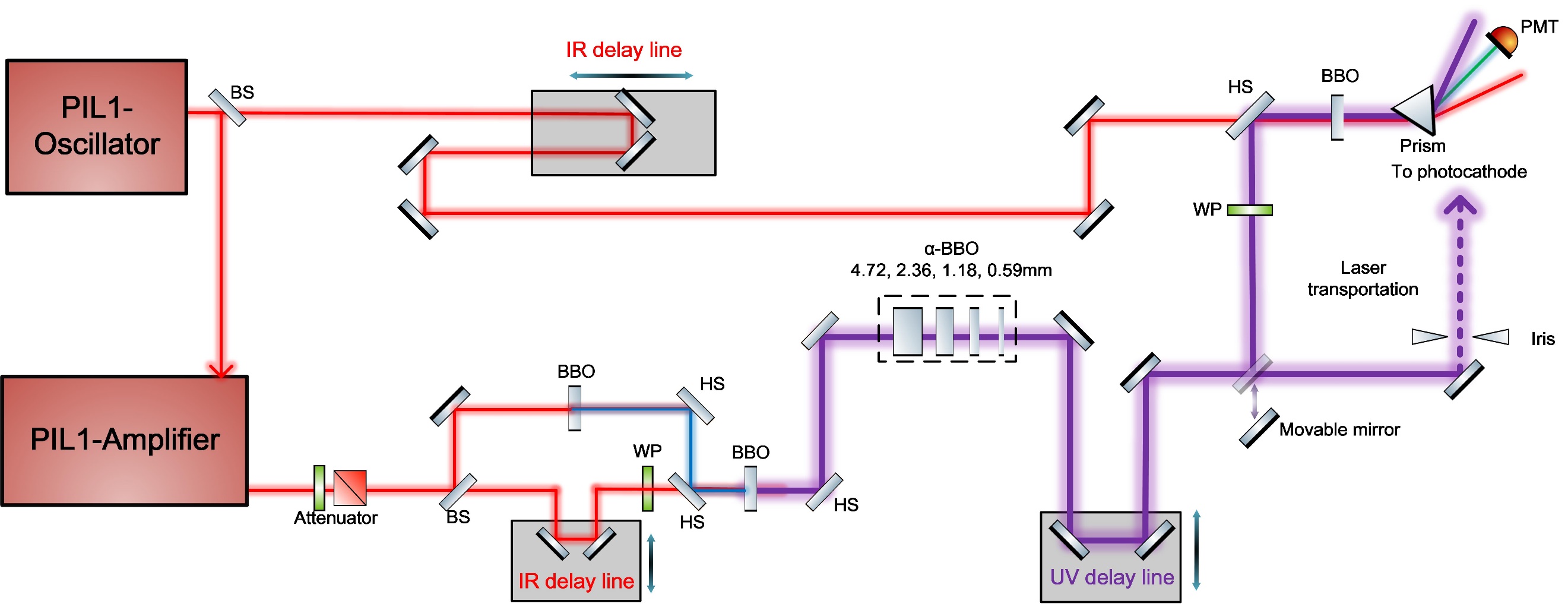


**Extended Data Fig. 2 Photoinjector drive laser generation and measurements.**


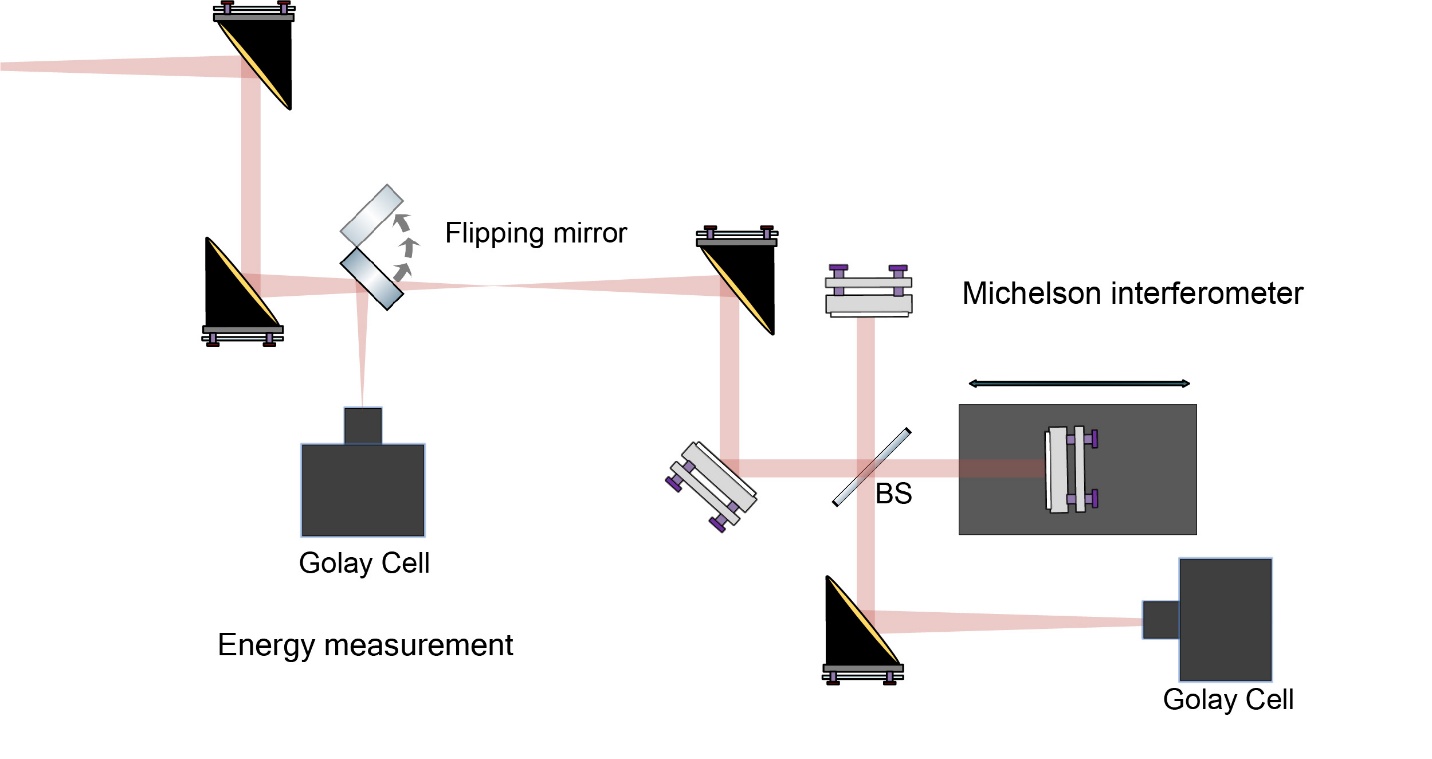


**Extended Data Fig. 3 THz measurement set-up**
